# P-1601. Seasonal Temperature Variations and COVID-19 Mortality: An Analysis of February and August Trends Across U.S. States

**DOI:** 10.1093/ofid/ofaf695.1780

**Published:** 2026-01-11

**Authors:** Elif N Ozkok, Zeynep Abul, Shelly Langshaw

**Affiliations:** Niceville High School, Niceville, FL; Northeastern University, BARRINGTON, Rhode Island; Fort Walton Beach High School, Niceville, Florida

## Abstract

**Background:**

COVID-19 deaths showed seasonal patterns across nine U.S. states from 2021–2022, with winter consistently deadlier than summer. Cold weather may prolong virus survival and weaken immunity, while summer heat drives people indoors, increasing transmission. This study compared February (winter) and August (summer) mortality rates to clarify these trends and guide public health strategies.

**Table 1:**
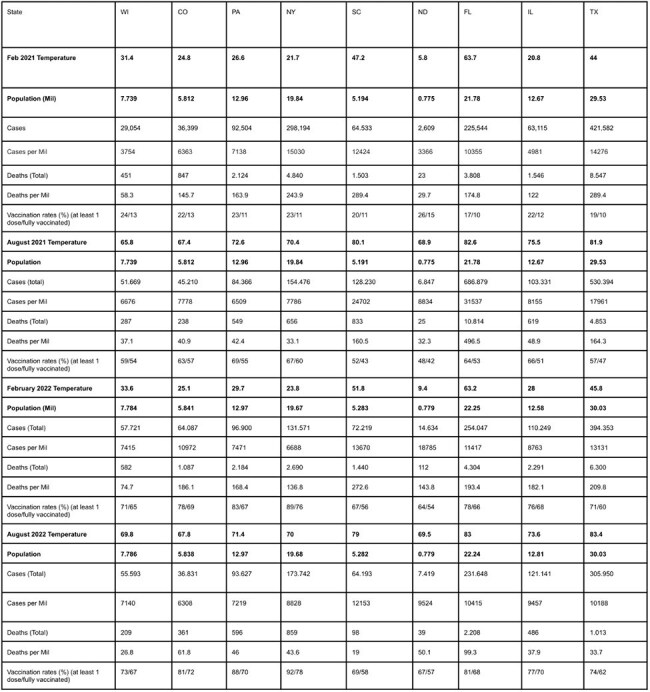
The focus on COVID-19 cases, deaths, and vaccination rates between February and August across two years

**Methods:**

COVID-19 deaths per million were compared between February and August using temperature data (Climate Reanalyzer) and mortality statistics (Worldometer). States ranged from northern (Wisconsin, North Dakota) to southern (Florida, Texas) climates. Vaccination rates (CDC, USAFacts) and population density were also analyzed.

**Results:**

Winter death rates were higher than summer in most states. Wisconsin’s rate fell from 58.7 per million in February 2021 to 37.1 in August; New York dropped from 243.9 to 33.1. In 2022, Texas had 209.8 deaths per million in February versus 33.7 in August; Florida saw 193.4 versus 99.3. Colder states did not always have higher winter deaths, suggesting other factors at play.

Vaccination rates rose sharply-Florida’s fully vaccinated population increased from 10% in February 2021 to 66% by February 2022-likely reducing summer deaths. However, winter surges persisted even as vaccines became widespread, hinting at cold weather’s direct impact. Differences in vaccination coverage (e.g., Florida’s 78% vs. Wisconsin’s 65% by 2022) also influenced outcomes (Table 1).

**Conclusion:**

COVID-19 mortality showed winter peaks, similar to other respiratory illnesses. The lack of clear north-south differences suggests factors like vaccination rates played a major role. Florida’s high summer deaths may reflect indoor behavior during heat waves, while lower summer mortality in 2022 reflected stronger immunity. Public health strategies should consider climate, healthcare capacity, and behavior when planning for seasonal surges.

**Disclosures:**

All Authors: No reported disclosures

